# Impending rupture of a chronic contained abdominal aortic aneurysm with a 14‐day history

**DOI:** 10.1002/ccr3.4737

**Published:** 2021-09-07

**Authors:** Takayuki Yamada, Susumu Ohwada, Kenzo Okauchi, Yutaka Hasegawa

**Affiliations:** ^1^ Asunaro Clinic Takasaki City Japan; ^2^ ASKOHWADA Consultation Clinic of Gastroenterology and Oncology Maebashi City Japan; ^3^ Department of Radiology Public Tomioka General Hospital Tomioka City Japan; ^4^ Cardiovascular Surgery Gunma Prefectural Cardiovascular Centre Maebashi City Japan

**Keywords:** abdominal aortic aneurysm, chronic contained rupture, impending rupture, low back pain, perspiration, prostration

## Abstract

This case of an impending abdominal aortic aneurysm rupture emphasizes impaired consciousness with low back pain. Family doctors must be attentive to a patient's physical findings and medical history, even if vital signs are normal at the initial visit.

## Graphical abstract

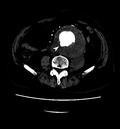

## 1 INTRODUCTION

A 73‐year‐old man, who, 10 days before admission, lost consciousness while driving, visited our clinic with severe back pain. His vital signs were normal, but findings suggested hypovolemic shock. Emergency surgery for an abdominal aortic aneurysm rupture saved his life. Physicians should be attentive to physical findings.

Low back pain in adults is common. Acute low back pain is caused by musculoskeletal disorders and generally improves to 90% of the pre‐disease condition within 6 weeks. Approximately 1% of back pain cases are due to cardiovascular diseases and require prompt and accurate diagnosis; the misdiagnosis of such cases can be fatal within a few days.^1^

We encountered a patient whose back pain had worsened for over 14 days. While driving with his wife 10 days earlier, he became confused and dizzy, took a wrong turn, and stepped on the accelerator pedal instead of the brake pedal. He barely escaped a car accident. His first examination was in a pre‐shock state, and the patient was transferred to the emergency room. The patient was diagnosed with an impending chronic containment rupture of an abdominal aortic aneurysm (AAA) and underwent emergency aortic replacement surgery. The symptoms leading to his visit were specific, and this case illustrates the importance of family physicians being attentive to physical findings at the first visit.

## 2 CASE PRESENTATION

A 73‐year‐old man with well‐controlled bronchial asthma presented to our clinic with a chief complaint of left low back pain and numbness due to radiation to the left low back and low extremities that had worsened more than 2 weeks earlier. He denied any history of abdominal pain, but had chronic back pain, which was usually relieved with use of over‐the‐counter medications, primarily non‐steroidal anti‐inflammatory drugs (NSAIDs). He had no history of hypertension or diabetes, but had been a chronic smoker (50 pack‐years) until 5 years prior. Ten days before visiting our office, he suddenly felt immense anxiety, became confused and dizzy while driving with his wife, drove in the wrong direction, and stepped on the gas pedal instead of the brake pedal. They narrowly escaped a serious car accident. His wife, thinking that her husband had suddenly developed dementia, immediately took him to a neurology clinic for cognitive evaluation. A cranial computed tomography (CT) scan showed normal findings, and a mini‐mental state test diagnosed dementia. His vague anxiety was relieved. However, the back pain worsened. It was augmented by walking, lying down, getting up, sitting, and semi‐crouching; over‐the‐counter medications had no effect. He walked to our clinic, spoke normally, and complained of severe back pain with pain in his left hip. On physical examination, the anal wink and left lumbar muscle signs were positive, the left lumbar paraspinal muscles were tender, and there were no sciatic nerve irritation symptoms. His vital signs were normal (blood pressure, 119/80 mmHg; heart rate, 83 beats/min; body temperature, 36.3°C; and SpO_2_, 95% room air). However, he was perspiring profusely, his hands were cold due to vasoconstriction, and he had tachypnea (respiratory rate = 20 breaths/min), suggesting a pre‐shock state. We diagnosed the patient as urgently needing to be transferred to the emergency hospital. We persuaded the emergency room physician of the Prefectural Cardiovascular Centre, who was initially reluctant to admit him because of his normal blood pressure, that the patient was in a pre‐shock state of weakness, perspiration, and tachypnea. Finally, he accepted the emergency transport of the patient, and the following vital signs were measured at the time of admission: 141/96 mmHg, heart rate 74 beats/min, SpO_2_ 96%, hematocrit 35.4%, and hemoglobin 11.6 g/dl.

**FIGURE 1:**
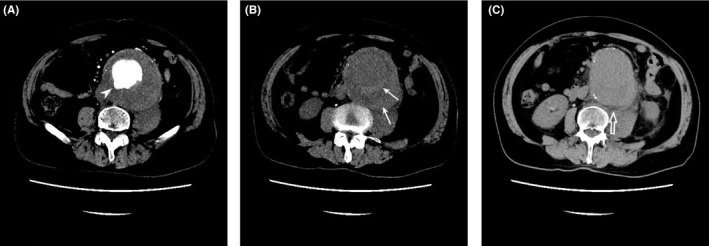
(A) A crescentic high‐density area in the intramural thrombus and penetrating atherosclerosing ulcer (PAU) of the aortic lumen posterior wall; (B) Left iliopsoas muscle compression with slight swelling; (C) Fatty opacity around the aorta and a thin, mildly dense fluid

Non‐contrast‐enhanced CT showed a juxtarenal aorto‐iliac abdominal aneurysm (AAA), maximum size 100 mm × 92 mm. Contrast‐enhanced CT demonstrated a crescentic high‐density area in the intramural thrombus and a penetrating atherosclerosing ulcer (PAU) of the aortic lumen posterior wall (Figure 1A, arrowhead). No extravasation of contrast material was documented, and non‐contrast‐enhanced CT showed fatty opacity around the aorta, thin mildly dense fluid (Figure 1B, arrow), and left iliopsoas muscle compression with slight swelling (Figure 1C, open arrow). These results indicated a mild leak or imminent rupture of an AAA. Endovascular aortic aneurysm repair (EVAR) was not carried out as it is not indicated for the juxtarenal and the impending rupture of the aorto‐iliac AAA.^2^, ^3^

An emergency laparotomy was applied and revealed that the posterior wall cavity of the abdominal aorta was ruptured. Emergency abdominal aortic replacement surgery with expanded polytetrafluoroethylene saved his life. The patient recovered well and did not have any major health issues or complications at the 8‐month follow‐up.

## 3 DISCUSSION

This case teaches us that a focused history and detailed physical examination are extremely important in primary care settings. Family or general physicians must be attentive to potentially fatal and urgent physical findings. In this case, the patient was a walk‐in and had a normal blood pressure and heart rate; however, his pre‐shock physical findings were an urgent sign; he was, therefore, transferred to the emergency room and underwent emergency aortic replacement surgery for a chronic AAA containment rupture.

First, despite the pre‐shock signs in our clinic, the compensatory circulatory state suggested decreased circulating blood volume due to gastrointestinal bleeding or vascular etiologies. However, he did not have any symptoms or history of acute or chronic upper or lower gastrointestinal bleeding. Therefore, vascular etiology most likely caused his pre‐shock state. Regarding his medical history, it is possible that his cognitive decline after he temporarily lost consciousness while driving with his wife 10 days prior to his visit was a sign of cerebral ischemia. Considering the cerebral ischemia due to decreased circulating blood volume and the worsening of chronic back pain, an AAA rupture or retroperitoneal hemorrhage due to malignancy was suspected. The fact that the patient visited our clinic 10 days later meant that the bleeding had spontaneously stopped, and he was on the verge of being in danger. An emergency laparotomy revealed a localized rupture of the posterior abdominal aorta covered by the retroperitoneal tissue.

Furthermore, since over‐the‐counter NSAIDs relieved his back pain, he was not concerned about the worsening of his back pain after more than 2 weeks. A retrospective review of the CT images showed mild lumbar spondylosis, which may have caused the chronic low back pain. Chronic low back pain is usually musculoskeletal in origin and generally improves in about 4 weeks. It is impossible to distinguish from the clinical symptoms and history whether the acute back pain was an exacerbation of chronic back pain or a new symptom. The presence of an iliopsoas sign during physical examination and the absence of a history of fever and abdominal pain can exclude inflammatory or infectious diseases as a cause of low back pain. These factors may provide clues to proceed with the diagnosis retrospectively. Worsening back pain and the failure of analgesics are a red flag, and retroperitoneal organs, such as the pancreas, kidneys, lymph nodes, bones, and muscles, should be examined using CT or magnetic resonance imaging (MRI).

Taking physical findings at the first visit as well as collecting clinical history are important for making a correct diagnosis as a primary physician. Despite recognition of the findings of a pre‐shock state, a physical examination of the chest and abdomen was not undertaken. If we had informed the emergency physician that the patient had a large pulsatile mass in the abdomen, the emergency physician would have accepted the transportation urgently.

In contrast, chronic AAA contained rupture can cause back pain. As shown by CT, crescentic signs indicated that the chronic thrombus was contained, and the PAU in the aortic lumen posterior wall indicated recurrent and imminent contained AAA rupture. The back pain, in this case, was possibly a sign of a chronic contained AAA rupture. It was unclear when this chronic contained AAA rupture would occur. The pre‐shock signs remaining at the time of the walk‐in could have suggested the recurrence of the contained AAA rupture, and the worsening of his back pain could have suggested continued blood penetration into the chronic contained AAA rupture. CT or MRI must be performed for patients with chronic low back pain for more than 3 months or with acute exacerbation of low back pain.

Among the risk factors for AAA, our patient did not have hypertension, a history of atherosclerosis, a familial history of AAA, or a high alcohol intake (≥30 g/day or ≥2 drinks/day). However, he was 75 years of age and had been a chronic smoker (50 pack‐years) who had quit smoking 5 years prior to admission.^4^, ^5^

Family or general physicians must keep these risk factors in mind to rapidly and correctly conduct differential diagnosis.

Finally, when reviewing the patient's history, characteristic medical history clues and positive physical findings can guide the differential diagnosis within this triage category of aneurysm ruptures. This case highlights the significance of impaired consciousness with low back pain and reminds us once again that a detailed physical examination and attention to signs of ruptures are essential in primary care settings.

## CONFLICT OF INTEREST

None declared.

## AUTHOR CONTRIBUTION

TY: Served as the diagnostician and first author. SO: Served as the supervisory doctor. KO: Served as the computed tomography diagnostician. YH: Served as the operator.

## ETHICAL APPROVAL

Ethics approval was not required for this study.

## Data Availability

The data that support the findings of this study are openly available.
